# Uric Acid and Cardiovascular Disease: An Update From Molecular Mechanism to Clinical Perspective

**DOI:** 10.3389/fphar.2020.582680

**Published:** 2020-11-16

**Authors:** Wei Yu, Ji-Dong Cheng

**Affiliations:** Department of Internal Medicine, Xiang’an Hospital of Xiamen University, Xiamen, China

**Keywords:** uric acid, cardiovascular disease, molecular mechanism, therapeutics, clinical prospect

## Abstract

Uric acid (UA) is the end product of purine nucleotide metabolism in the human body. Hyperuricemia is an abnormally high level of UA in the blood and may result in arthritis and gout. The prevalence of hyperuricemia has been increasing globally. Epidemiological studies have shown that UA levels are positively correlated with cardiovascular diseases, including hypertension, atherosclerosis, atrial fibrillation (AF), and heart failure (HF). Hyperuricemia promotes the occurrence and development of cardiovascular diseases by regulating molecular signals, such as inflammatory response, oxidative stress, insulin resistance/diabetes, endoplasmic reticulum stress, and endothelial dysfunction. Despite extensive research, the underlying molecular mechanisms are still unclear. Allopurinol, a xanthine oxidase (XO) inhibitor, has been shown to improve cardiovascular outcomes in patients with HF, coronary heart disease (CHD), type 2 diabetes (T2D), and left ventricular hypertrophy (LVH). Whether febuxostat, another XO inhibitor, can improve cardiovascular outcomes as well as allopurinol remains controversial. Furthermore, it is also not clear whether UA-lowering treatment (ULT) can benefit patients with asymptomatic hyperuricemia. In this review, we focus on the latest cellular and molecular findings of cardiovascular disease associated with hyperuricemia and clinical data about the efficacy of ULT in patients with cardiovascular disease.

## Introduction

Uric acid (UA) is the end product of purine metabolism in higher animals, such as humans and great apes. Under physiological conditions, UA synthesis and excretion are balanced in the body. Once this balance is disturbed, it leads to hyperuricemia. Normally, male UA levels greater than 7 mg/dL or female UA levels greater than 6 mg/dL are considered to be hyperuricemia (Hao et al., 2019). However, Virdis et al. confirmed that the threshold of UA level increased total mortality (4.7 mg/dL) and cardiovascular mortality (5.6 mg/dL) risk, which was significantly lower than clinical diagnostic criteria (Virdis et al., 2020). With increasingly unhealthy lifestyles, the incidence of hyperuricemia is increasing, and it has become the “fourth highest” after hypertension, hyperglycemia, and hyperlipidemia. It is estimated that the total number of patients with hyperuricemia was 170 million in China (Hao et al., 2019) and 32.5 million in the United States (Singh et al., 2019). Large-scale clinical studies on the relationship between serum uric acid (sUA) and cardiovascular diseases started from the Framingham Heart Study in the 1980s, the results of which indicate that UA does not have a causal role in the development of coronary heart disease (CHD), death from cardiovascular disease or all causes (Culleton et al., 1999). Recent epidemiological studies show that hyperuricemia may be involved in hypertension, diabetes, atherosclerosis, chronic kidney disease, and atrial fibrillation (AF) as well as the occurrence of cardiovascular events (Kuwabara et al., 2017a; Kuwabara et al., 2017b; Kuwabara et al., 2017c; Kuwabara et al., 2018a; Kuwabara et al., 2018b; Maruhashi et al., 2018). Experimental studies show that hyperuricemia promotes the occurrence and development of cardiovascular diseases by regulating molecular signals, such as inflammatory response (Xiao et al., 2015; Johnson et al., 2018; Lu et al., 2019), oxidative stress (Li et al., 2018), insulin resistance (Zhi et al., 2016), endothelial dysfunction (Maruhashi et al., 2018), and endoplasmic reticulum stress (Li P. et al., 2016; Yan et al., 2018).

An increasing number of clinical studies show that allopurinol can improve cardiovascular outcomes in patients with heart failure (HF), CHD, type 2 diabetes (T2D), and left ventricular hypertrophy (LVH) (Doehner et al., 2002; Farquharson et al., 2002; George et al., 2006; Noman et al., 2010; Rekhraj et al., 2013; Szwejkowski et al., 2013). Compared to allopurinol, whether febuxostat improves cardiovascular outcomes remains controversial (White et al., 2018; Zhang et al., 2018; Chen et al., 2019). Furthermore, whether UA-lowering treatment (ULT) is beneficial to patients with asymptomatic hyperuricemia is uncertain because these patients are often associated with a variety of risk factors (such as old age, chronic kidney disease, cardiovascular disease, obesity, metabolic syndrome, alcohol, or smoking habits, etc.) but without identified diseases. In addition, UA is an effective antioxidant in cardiovascular and neurodegenerative diseases (Huang et al., 2017), making the relationship between UA and cardiovascular disease more complicated.

This review focuses on the underlying mechanisms of cardiovascular disease associated with hyperuricemia and the efficacy of ULT in patients with cardiovascular disease.

## UA and its Related Molecular Mechanism

### UA and Oxidative Stress

UA is the final product of dietary purines. Existing evidence suggests that UA has a dual-face role in some cardiovascular and cerebrovascular diseases. On the one hand, UA has antioxidant activity and can scavenge reactive oxygen species (ROS). As one of the main endogenous antioxidants in the human body, UA contributes up to 60% of the plasma antioxidant capacity, which can protect cells from oxidative stress (Ames et al., 1981; Sautin and Johnson, 2008). The molecular mechanism of the antioxidation of UA are as follows: 1. It reacts directly with hydroxyl radicals, peroxynitrite, nitric oxide, and hydrogen peroxide, etc., forming stable intermediates (Gersch et al., 2009); 2. it cooperates with superoxide dismutase to scavenge oxygen radicals (Waring et al., 2001); 3. it chelates with metal ions (Davies et al., 1986); and 4. it inhibits the peroxynitrite-induced protein nitrification, protein, and lipid peroxidation (Whiteman et al., 2002). On the other hand, UA can promote oxidation activity in cells, which may be related to oxygen free radicals produced by ROS. The pro-oxidation mechanisms of UA include 1. reducing the production of nitric oxide in arterial endothelial cells and inhibiting vasodilation (Papezikova et al., 2013); 2. inhibiting the synthesis of adiponectin in adipocytes; 3. damaging the tricarboxylic acid cycle and fatty acid β oxidation; 4. activating the renin-angiotensin system, stimulating the proliferation of vascular smooth muscle cells and the production of angiotensin II (Ang II) (Corry et al., 2008); and 5. generating a chronic inflammatory reaction (Inaba et al., 2013). The dual effects of UA on anti- and pro-oxidants may be closely related to the xanthine oxidase (XO) activity in circulation. Several studies have associated the involvement of XO activity, a source of UA and ROS, to pro-oxidative and pro-inflammatory effects during pathological conditions (Boban et al., 2014; Kushiyama et al., 2016; Klisic et al., 2018).

**Figure 1:**
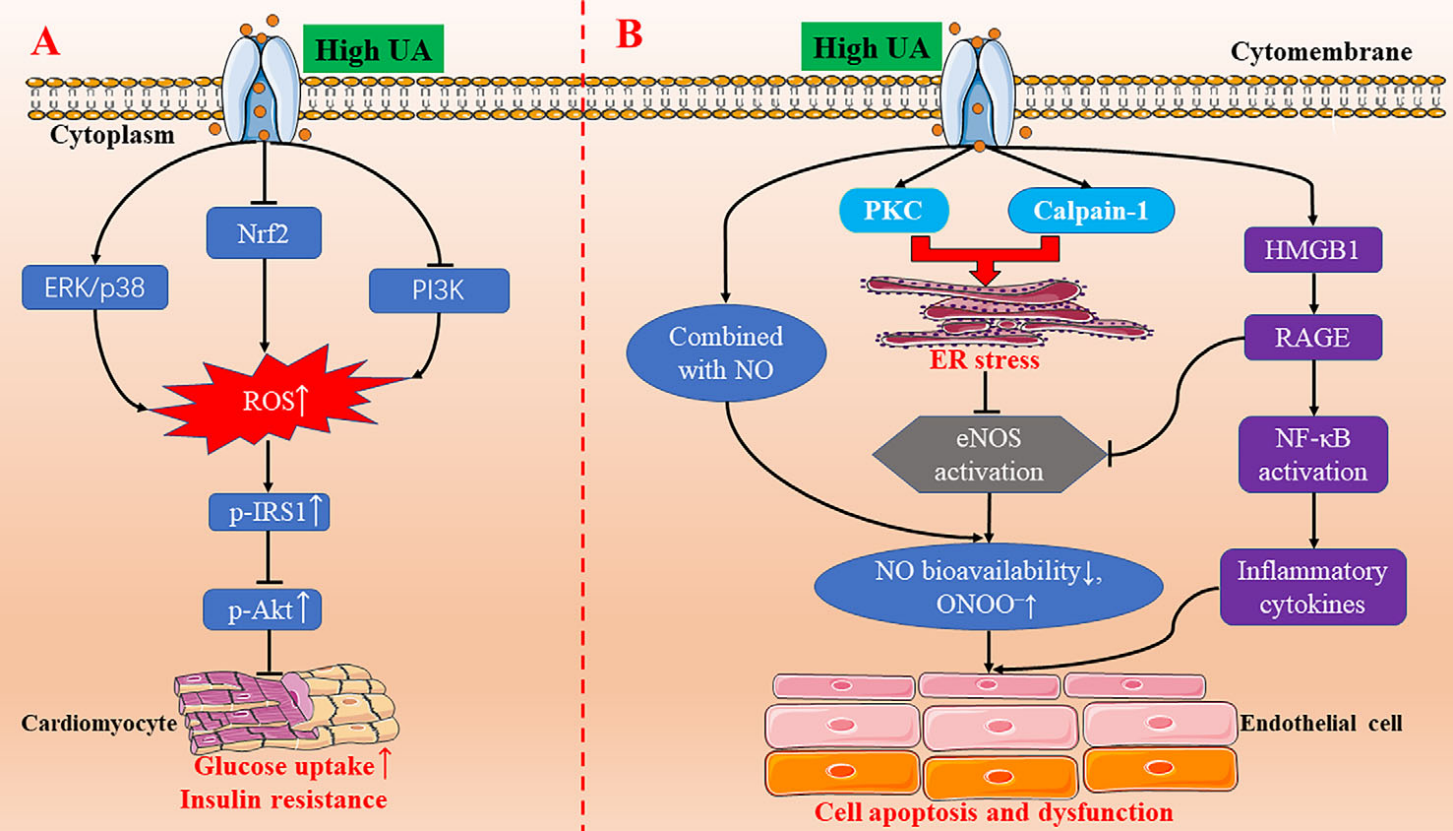
The molecular mechanisms of high UA-inducing oxidative stress, insulin resistance, endoplasmic reticulum stress, and endothelial dysfunction. **(A)** High UA activating the ERK/p38 signal pathway and inhibiting the Nrf2 and PI3K/Akt signal pathway, leading to an increase in the production of ROS. Increased ROS triggers IRS1/Akt phosphorylation to induce insulin resistance in cardiomyocytes. **(B)** High UA induces endothelial cell apoptosis and endothelial dysfunction through endoplasmic reticulum stress and the HMGB1/RAGE pathway. eNOS, endothelial nitric oxide synthase; ER, endoplasmic reticulum; ERK, extracellular signal-regulated kinase; HMGB1, high mobility group box chromosomal protein 1; NF-κB, nuclear factor κB; NO, nitric oxide; Nrf2, NF-E2-related factor 2; p-AKT, phospho-Akt; PI3K, phosphatidylinositol 3-kinase; p-IRS1, phospho-insulin receptor substrate 1; PKC, protein kinase C; RAGE, receptor for advanced glycation end products; ROS, reactive oxygen species; UA, uric acid.

In recent years, a lot of progress has been made on the relationship between UA and oxidative stress and its molecular mechanism. The physiological concentration of UA decreased oxidative stress–induced malondialdehyde and protein carbonyl contents, promoted superoxide dismutase (SOD) activity, and inhibited the formation of ROS in chicken embryo cardiomyocytes (Sun et al., 2017). The underlying mechanism was the NF-E2-related factor 2 (Nrf2) signal pathway (Sun et al., 2017). In contrast, treated with a high concentration of UA (1200 µM), the Nrf2 signaling pathway was inhibited, malondialdehyde and protein carbonyl contents were increased, and SOD activity was decreased (**Figure 1A**) (Sun et al., 2017). Our research team found that high UA inhibited the viability of H9c2 cardiomyocytes and increased the production of ROS (Li et al., 2018). Pretreatment with ROS scavenger (N-acetyl-L-cysteine) and extracellular signal-regulated kinase (ERK) inhibitor (PD98059) reversed the decrease of cell viability induced by high UA (Li et al., 2018). Further studies show that high UA-induced ROS may be closely related to ERK/p38 activation and phosphatidylinositol 3-kinase (PI3K)/Akt inhibition (**Figure 1A**) (Li et al., 2018). In addition, UA has a neuroprotective effect on dopaminergic neurons in Parkinson's disease mice, which may be related to Nrf2-ARE signal activation, reduction of oxidative damage, and neuroinflammation (Huang et al., 2017).

In short, previous studies have confirmed that UA has both antioxidant and pro-oxidant effects in vivo. This dual effect of UA has also been confirmed in cardiomyocytes. The latest experimental studies demonstrate that the antioxidation of UA may be involved in regulating the Nrf2-ARE, ERK/p38, and PI3K/Akt signaling pathways.

### UA and Inflammatory Response

**Figure 2:**
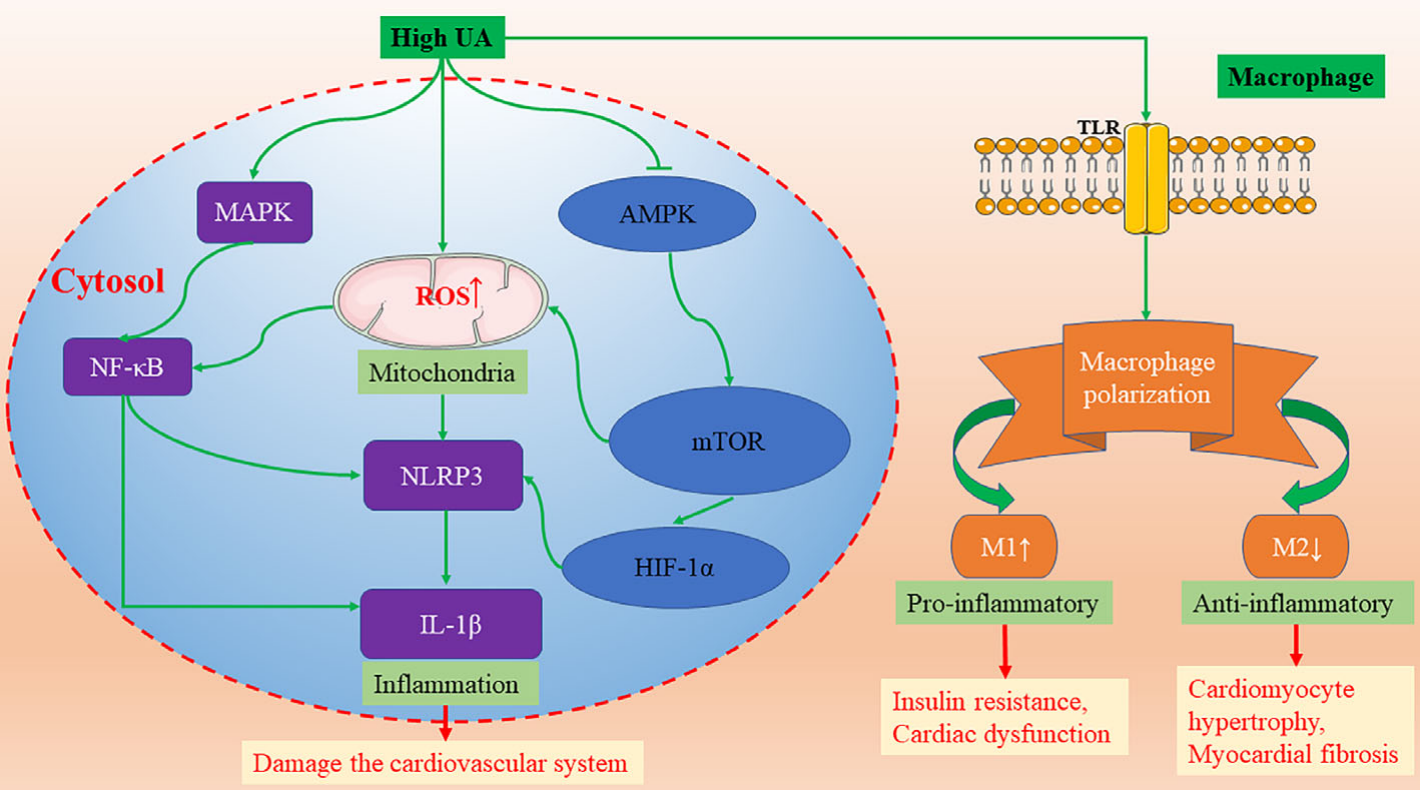
The molecular mechanisms of high UA activating NLRP3-inflammasomes and promoting macrophage M1/M2 polarization. AMPK, AMP-activated protein kinase; HIF-1α, hypoxia-inducible factor-1α; IL-1β, interleukin-1β; MAPK, mitogen activated protein kinases; mTOR, mammalian target of rapamycin; NF-κB, nuclear factor κB; NLRP3, nod-like receptor protein 3; TLR, Toll-like receptors; UA, uric acid.

UA has been a focus of research not only for its role in oxidative stress, but also for its relation to various inflammatory diseases. Atherosclerosis is a chronic immuno-inflammatory cardiovascular disease. A large body of evidence suggests that elevated UA levels are strongly associated with the occurrence and development of atherosclerosis. High intracellular UA concentrations promote the expression of inflammatory markers, such as nuclear factor κB (NF-κB), growth factors, vasoconstrictive substances (Ang II, thromboxane, and endothelin-1), and chemokines via activating mitogen-activated protein kinases (MAPK) (**Figure 2**) (Xiao et al., 2015; Johnson et al., 2018). Additionally, hyperuricemia promoted macrophage M1/M2 polarization, which could be reversed by ULT (Jia et al., 2015). In the development of obesity and cardiorenal disease, UA tends to enhance the pro-inflammatory response of M1 and inhibit the anti-inflammatory response of M2 (Aroor et al., 2013). M1 macrophages secrete inflammatory cytokines, leading to insulin resistance and cardiac dysfunction (Aroor et al., 2013). On the contrary, M2 macrophages secrete interleukin-10 (IL-10), which inhibits cardiomyocyte hypertrophy and myocardial fibrosis (**Figure 2**) (Bene et al., 2014). Furthermore, in a randomized, open, parallel-controlled study involving 176 patients with T2D and asymptomatic hyperuricemia, allopurinol effectively lowered sUA, improved insulin resistance, reduced serum high-sensitivity C-reactive protein (hs-CRP) level, decreased carotid intima-media thickness, and ameliorated the exacerbation of atherosclerosis (Liu et al., 2015).

The activation of inflammasomes plays a critical role in a variety of chronic inflammatory diseases. Recently, many studies have found that sUA can effectively activate inflammasomes in the cardiovascular system. The continuous activation of inflammasomes aggravates the inflammatory response and damages the cardiovascular system. Studies by Wang et al. report that UA can activate nod-like receptor protein 3 (NLRP3) inflammasomes and damage mitochondria, resulting in cellular damage of H9c2 cells (Wang et al., 2018). In addition, several studies also show that UA activates the NLRP3 inflammasome and induces interleukin-1β (IL-1β) release in a variety of cells, including monocytes, macrophages, vascular smooth muscle cells, and endothelial cells (Martinon et al., 2006; Matias et al., 2015; Alberts et al., 2019; Kim et al., 2019; Li et al., 2019; Yin et al., 2019). The molecular mechanism of UA-induced NLRP3/IL-1 β activation is through NF-κB activation and mitochondrial ROS (mROS) (**Figure 2**). Consistent with the latest research published by Kimura et al., UA promotes the secretion of IL-1β mediated by NLRP3 inflammasomes via regulating the AMP-activated protein kinase (AMPK)-mammalian target of rapamycin (mTOR) mROS and hypoxia-inducible factor-1α (HIF-1α) pathway in human peripheral blood mononuclear cells (**Figure 2**) (Kimura et al., 2020). In mice treated with uricase gene transfer and XO inhibitor, however, the decrease of UA level promoted the activation of AMPK, and the formation of atherosclerotic plaque was inhibited (Kimura et al., 2020).

In conclusion, UA has been shown to promote inflammation in a variety of cells. Hyperuricemia can promote the development of atherosclerosis by regulating inflammatory signal pathways, such as NLRP3-inflammasomes, macrophage M1/M2 polarization, and hs-CRP, and ULT can significantly reverse the formation of atherosclerotic plaques. Inhibition of UA-induced NLRP3 inflammasome activation may be a new therapeutic target for atherosclerosis.

### UA and Insulin Resistance/Diabetes

Insulin resistance is closely related to diabetes, obesity, hyperlipidemia, hypertension, hyperuricemia, and other metabolic disorders. Current studies confirm that oxidative stress and inflammation may be the pathophysiological basis of insulin resistance. Hyperuricemia can promote oxidative stress in many cell lines. The rise of ROS level can induce insulin resistance. Oxidative stress may be the cause of insulin resistance–related cardiovascular complications because overgenerated ROS and insulin resistance may lead to cardiac dysfunction (Ritchie, 2009). Our team's research shows that high UA can increase ROS production and inhibit insulin-induced glucose uptake in H9c2 and primary cardiomyocytes, and N-acetyl-L-cysteine pretreatment can reverse the inhibitory effect of high UA on glucose uptake (Zhi et al., 2016). The molecular mechanism may be that high UA increases the phosphorylation of insulin receptor substrate 1 (IRS1) and inhibits the phosphorylation of Akt, which was blocked by N-acetyl-L-cysteine (**Figure 1A**) (Zhi et al., 2016). Hence, high UA can induce insulin resistance in cardiomyocytes in vitro and in vivo.

Clinical studies have shown a link between hyperuricemia and diabetes (Kodama et al., 2009; van der Schaft et al., 2017); however, it is controversial whether hyperuricemia plays a causal role in diabetes. Recently, Lu et al. confirmed that there is no causal relationship between hyperuricemia and diabetes (Lu et al., 2020). High UA accelerates but does not cause diabetes because UA itself is insufficient to induce diabetes although it can damage glucose tolerance, leading to insulin resistance (Lu et al., 2020). Although the mechanism of high UA–induced myocardial insulin resistance has not been fully elucidated, it might be a novel potential mechanism of hyperuricemic-related cardiovascular disease.

### UA and Endoplasmic Reticulum Stress

Cardiomyocyte apoptosis is one of the pathogeneses of myocardial anatomical reconstruction. Oxidative stress and endoplasmic reticulum stress are the key factors promoting apoptosis, which are involved in the pathogenesis of many diseases, including cardiovascular diseases. UA triggers oxidative stress and endoplasmic reticulum stress to signal the network to induce endothelial dysfunction via activating the protein kinase C (PKC) pathway in human umbilical vein endothelial cells (HUVECs) (Li P. et al., 2016). The latest research also demonstrates that UA can induce cardiomyocyte apoptosis in vitro and in vivo, and its molecular mechanism might be through the activation of calpain-1 and the endoplasmic reticulum stress signaling pathway (**Figure 1B**) (Yan et al., 2018). So far, the studies on UA-induced endoplasmic reticulum stress is scarce, but it is certain that there will be more research to clarify the relationship between the two in the future.

### UA and Endothelial Dysfunction

Endothelial cells secrete a variety of vasoactive substances, including vasodilators (nitric oxide, prostaglandin I_2_, endothelium-derived hyperpolarizing factor, etc.) and vasoconstrictors (endothelin-1, thrombin A2, Ang II, etc.) (Vane et al., 1990; Maruhashi et al., 2018). Vasodilator nitric oxide plays a key role in the development of atherosclerosis. The increase of UA level in cells directly combined with nitric oxide, which resulted in the decrease of nitric oxide bioavailability and the increase of peroxynitrite (ONOO^–^) (Maruhashi et al., 2018). ONOO^–^ is a strong oxidant, which can cause DNA damage, cell death, and lipid peroxidation (Maruhashi et al., 2018). In addition, Li et al. found that high UA-induced HUVEC apoptosis and endothelial dysfunction is through PKC-dependent endothelial nitric oxide synthase (eNOS) phosphorylation and endoplasmic reticulum stress, reducing eNOS activity and nitric oxide production (Li P. et al., 2016). Furthermore, a recent study also indicates that high UA inhibits eNOS expression and nitric oxide production in HUVECs, increases high mobility group box chromosomal protein 1 (HMGB1)/receptor for advanced glycation end products (RAGE) expression, activated NF-κB, and increased the inflammatory cytokine levels (Cai et al., 2017). All these results provide new insight into the mechanisms of UA-induced endothelial dysfunction (**Figure 1B**).

Endothelial dysfunction plays a critical role in the development and progression of atherosclerosis, leading to serious cardiovascular events. Experimental studies show that high UA aggravates the inflammation response and oxidative stress and, thus, leads to endothelial dysfunction (Xiao et al., 2015; Johnson et al., 2018; Li et al., 2018; Lu et al., 2019). Additionally, hyperuricemia-induced endothelial dysfunction is also confirmed in many clinical studies (Mercuro et al., 2004; Kato et al., 2005; Ho et al., 2010; Tomiyama et al., 2011; Sincer et al., 2014). All these experiments and clinical studies show that UA is not only a biomarker of cardiovascular risk, but also a causal risk factor of endothelial dysfunction (Katsiki and Mikhailidis, 2015). Notably, Borgi et al. found that ULT was not associated with endothelial dysfunction improvement in nonhypertensive, overweight/obese individuals (Borgi et al., 2017). Therefore, the hypothesis of the causal relationship between UA and endothelial dysfunction needs further study.

### UA and Cardiovascular Disease

#### UA and Hypertension

Elevated sUA is strongly associated with hypertension, but the mechanism is not clear. Hyperinsulinemia induced by insulin resistance enhances renal sodium reabsorption, which may lead to hypertension (Forman et al., 2009; Battelli et al., 2018). Endothelial dysfunction caused by oxidative stress also plays a key role in the development of hypertension, kidney disease, and cardiovascular disease (Mortada, 2017). Studies have shown that UA significantly increased the production of ROS and Ang II in human endothelial cells (Yu et al., 2010).

In recent years, numerous studies have demonstrated the link between sUA and hypertension. In a prospective randomized study of 5748 healthy adolescents, a 10-year follow-up showed that elevated sUA was closely associated with hypertension and metabolic syndrome (Sun et al., 2015) Recently, a retrospective cohort study of 3584 prehypertensive patients also showed that increased sUA was a strong risk marker for developing hypertension from prehypertension (Kuwabara et al., 2018b). In another large-scale meta-analysis of 55,607 subjects in 18 prospective cohort studies, it was found that, for every 1 mg/dl increase in sUA, the incidence of hypertension increased by 13% (Grayson et al., 2011). This result is consistent with the result recently published by Bjornstad et al. that higher baseline sUA independently increased the risk of incident hypertension (hazard ratio 1.19, per 1 mg/dL increase in sUA) (Bjornstad et al., 2019). Arterial stiffness and inflammation may be involved in the risk of development of hypertension associated with hyperuricemia (Tomiyama et al., 2018), but this result is inconsistent with Cicero et al., who show that sUA is significantly correlated to hypertension and carotid intima–media thickness but not to aortic stiffness (Cicero et al., 2014). Interestingly, recently published experimental studies do not seem to be consistent with the above epidemiological and clinical studies. Chen et al. show that UA level is not positively correlated with blood pressure (BP) in people with early Parkinson's disease (Chen et al., 2018). In addition, the increase of UA level was also not related to the change of BP, cardiac morphology, and heart dysfunction in a *Uricase/Uox* knockout male mouse model (Chen et al., 2018).

Likewise, this close relationship between UA and hypertension also exists in pregnant women. The level of sUA in pregnant women is higher than that in normal, nonpregnant women, and the incidence of hypertensive disorders of pregnancy is significantly increased in pregnant women with hyperuricemia (Sakr et al., 2020). The mechanisms of increased UA level during pregnancy may be related to the decrease of estrogen concentration, the increase of high purine food intake, and the increase of XO activity. Increased UA level strongly correlated to the severity of preeclampsia and higher risk of gout in later life (Wang I. K. et al., 2016; Khaliq et al., 2018).

**Figure 3:**
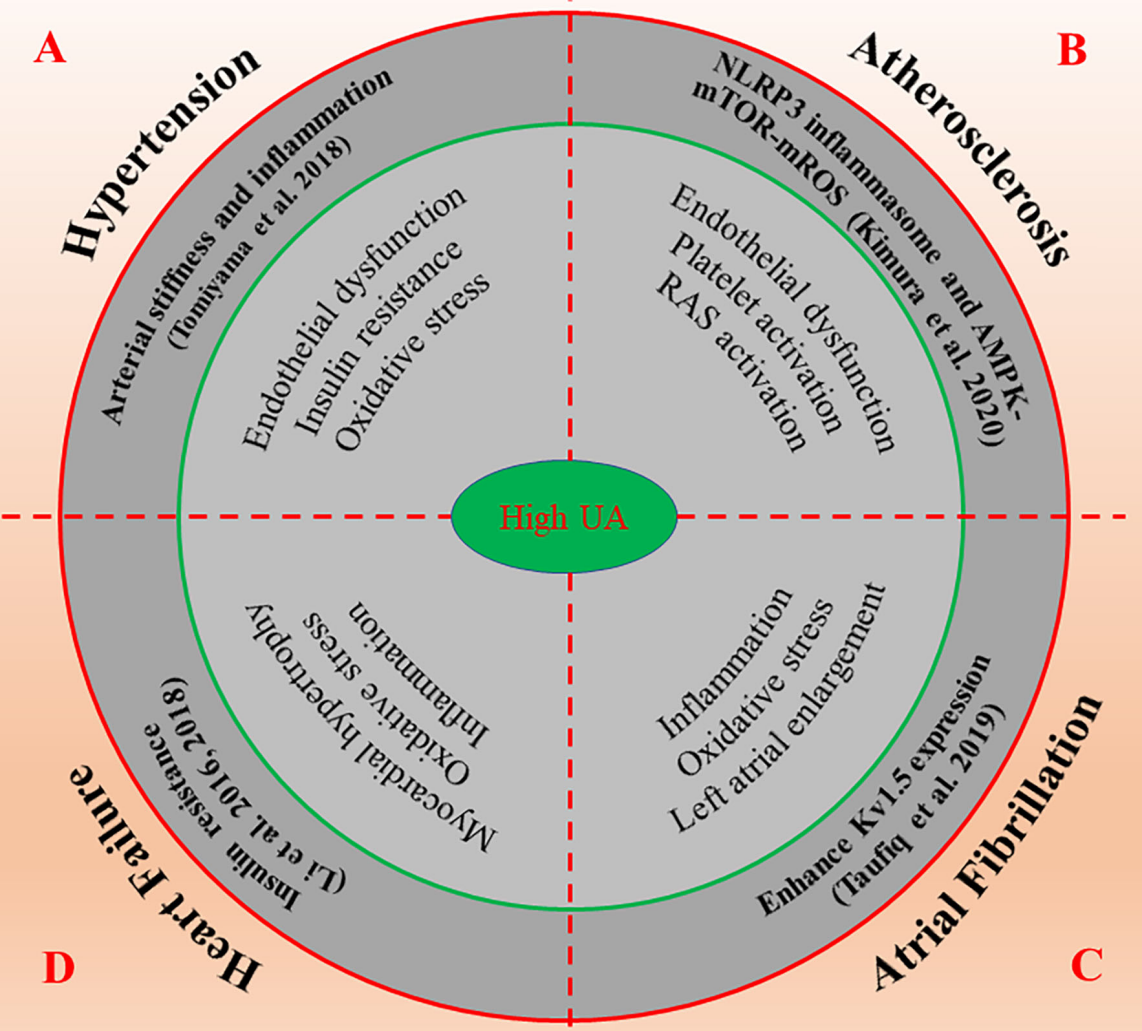
The possible molecular mechanisms of high UA promote the occurrence and development of cardiovascular diseases. High UA regulates numerous molecular signals such as inflammation, oxidative stress, insulin resistance, and endothelial dysfunction, thus affects the progression and prognosis of cardiovascular diseases including hypertension **(A)**, atherosclerosis **(B)**, atrial fibrillation **(C)** and heart failure **(D)**. AMPK, AMP-activated protein kinase; mROS, mitochondrial ROS; mTOR, mammalian target of rapamycin; NLRP3, nod-like receptor protein 3; RAS, renin-angiotensin system; UA, uric acid.

In conclusion, sUA is closely correlated with BP, but a consistent effect has not been observed in ananimal model and human studies. Fortunately, the recent published results by Johnson et al. give us an answer. After treatment with pegloticase, sUA remained at a low level in hyperuricemia patients, and its systolic and diastolic BP decreased significantly, which was independent of changes in renal function (Johnson et al., 2019). These findings give us reasons to believe that there may be a causal link between hyperuricemia and hypertension (**Figure 3A**).

### UA and CHD

Atherosclerosis is the most common cardiovascular disease with the highest morbidity and mortality. The possible mechanisms of UA induced CHD may include the following (Wu et al., 2012): 1. Vascular endothelial damage of large and micro vessels: urate is easy to deposit in the vascular wall and stimulate the proliferation of vascular smooth muscle cells; high UA can activate the renin-angiotensin system, inhibit nitric oxide production, and induce endothelial cell dysfunction (Corry et al., 2008; Sautin and Johnson, 2008; Papezikova et al., 2013). 2. UA can cause platelet activation, adhesion, and aggregation. 3. Hyperuricemia participates in the production of many inflammatory mediators (such as interleukin, C-reactive protein, etc.) (Inaba et al., 2013). 4. Hyperuricemia can increase the production of oxygen free radicals, which can cause low-density lipoprotein peroxidation, damage endothelial cells, promote vascular smooth muscle and intimal hyperplasia, etc. (Whiteman et al., 2002). 5. High UA can directly cause low-density lipoprotein oxidation (Wu et al., 2012).

A prospective cohort study of 457,915 subjects without cardiovascular disease found that UA concentrations >7.0 mg/dl significantly increased the risk of CHD in the general population (mainly adult women) (Braga et al., 2016). Another study suggested that sUA > 8 mg/dl was independently associated with three vessel diseases: coronary artery, HF, and LV enlargement in Chinese patients with early-onset CHD (Dai et al., 2015). In a meta-analysis of 12,677 subjects, elevated sUA level was found to be significantly associated with poor prognosis (all-cause mortality, cardiovascular mortality, and hospitalization for HF) in patients with LV dysfunction, HF, or both after myocardial infarction (MI) (von Lueder et al., 2015). sUA can be used as a low-cost and widely used biomarker to evaluate the clinical risk stratification of patients with MI. According to a systematic review of 29 prospective cohort studies involving 958,410 subjects, for every 1 mg/dl increase in sUA level, the mortality rate of CHD was 1.02 in men and 2.44 in women (Li M. et al., 2016). This result is consistent with the result published by Wang et al. that increased sUA levels are closely related to the risk of cardiovascular and all-cause mortality in people with suspected or definite CHD (Wang R. et al., 2016). For every 1 mg/dl increase in sUA, cardiovascular and all-cause mortality increased by 12% and 20%, respectively (Wang R. et al., 2016).

Although many clinical studies confirm that elevated sUA levels are strongly and independently associated with the occurrence and development of coronary atherosclerosis, the severity of coronary artery lesions and cardiovascular and all-cause mortality, the molecular mechanism of UA promoting atherosclerosis is still unclear. Plaque morphology, endothelial dysfunction, oxidative stress, and inflammation play a critical role in the development and progression of atherosclerosis, leading to serious cardiovascular events (Prasad et al., 2017). Optical coherence tomography showed a significant increase in plaque rupture in patients with sUA > 8.0 mg/dl (Kobayashi et al., 2018). Recently, Kimura et al. demonstrated that UA promotes the secretion of IL-1β mediated by NLRP3 inflammasomes via regulating the AMPK-mTOR mROS and HIF-1α pathway in human peripheral blood mononuclear cells (Kimura et al., 2020). In mice treated with uricase gene transfer and XO inhibitor, however, the decrease of UA level promoted the activation of AMPK and inhibited the formation of atherosclerotic plaque (Kimura et al., 2020). In addition, the coronary artery calcium (CAC) score can reflect total atherosclerotic burden, which is another effective index to predict future CVD events. Jun et al. evaluated the correlation between CAC and sUA in 9297 subjects by multidetector CT (Jun et al., 2018). The results show that sUA level is an independent predictor for development of moderate CAC in generally healthy adults (Jun et al., 2018).

In brief, hyperuricemia is a potential risk factor for CHD. The elevated sUA may lead to endothelial dysfunction through inflammation and oxidative stress and the formation of unstable lipid plaque in the coronary artery, which eventually leads to the occurrence of atherosclerosis. Therefore, lowering the level of UA is crucial in the prevention and treatment of CHD (**Figure 3B**).

### UA and AF

AF is the most common heart rhythm disorder, and its major risk factors include advanced age, hypertension, obesity, diabetes, HF, valvular heart disease, and MI. In recent years, numerous clinical studies have confirmed that hyperuricemia is associated with AF after adjusting for various cardiovascular risk factors, and the correlation is stronger in women than in men (Liang et al., 2016; Zhang et al., 2016; Chen et al., 2017; Kuwabara et al., 2017c; Mantovani et al., 2018; Pak et al., 2018; Black-Maier and Daubert, 2019; Lin et al., 2019). The bases for sex-related differences in high UA and AF or CHD remains unknown. High UA has been associated with endothelial dysfunction in postmenopausal women, suggesting that high UA could be an independent risk factor for cardiovascular disease, including AF and CHD, particularly among postmenopausal women (Maruhashi et al., 2013; Lin et al., 2019).

In addition, sUA was also an independent risk factor of LA thrombus in patients with nonvalvular AF or with mitral stenosis in sinus rhythm (Tang et al., 2014; Ozturk et al., 2015). UA level is negatively correlated with the systolic function of the lower left atrial appendage (LAA) (Celik et al., 2015). It can provide prognostic information for long-term thromboembolic events in patients with AF (Celik et al., 2015). There are also studies suggesting that hyperuricemia is associated with an increased risk of AF recurrence after catheter ablation (Canpolat et al., 2014). In a recent meta-analysis involving 1298 patients with AF, however, elevated sUA was not associated with the risk of AF recurrence after catheter ablation (Zhao et al., 2016).

The mechanisms of high UA-promoted AF and thrombosis are not clear. A possible explanation for this association is that high UA induces oxidative stress and inflammation. Moreover, hyperuricemia was independently associated with increased left atrial diameter (Tamariz et al., 2011; Black-Maier and Daubert, 2019). Left atrial enlargement is closely related to the occurrence of AF and thrombosis. Furthermore, UA increases Kv1.5 protein expression by activating ERK and oxidative stress in mouse atrial myocytes (HL-1 cells) so as to enhance the ultra-rapid delayed-rectifier K^+^ channel currents and shorten the action potential duration (Maharani et al., 2015). Additionally, the molecular mechanism of the UA-induced enhancement of Kv1.5 expression also may be attributed to enhanced phosphorylation of Akt and heat shock factor 1 (HSF1), which leads to increased expressions of heat shock protein 70 (Hsp70) (Taufiq et al., 2019).

Although the relationship between hyperuricemia and AF has been widely recognized, the mechanism of occurrence/maintenance of AF caused by hyperuricemia has not been fully elucidated. The available evidence suggests that the UA-induced enhancement of Kv1.5 expression may be a novel mechanism. However, whether intervening can reduce the risk of AF remains unclear. It is clear that prospective intervention studies are needed in the future to demonstrate whether reducing the level of sUA is important for the prevention of AF (**Figure 3C**).

### UA and HF

HF is the end stage of most cardiovascular diseases, which is closely related to hypertension, MI, AF, and valvular heart disease. UA may play an important role in HF. A large number of studies (Vaduganathan et al., 2014; Palazzuoli et al., 2017; Pavlusova et al., 2019) and meta-analyses (Huang et al., 2014) have evaluated the relationship between sUA and the risk and adverse outcome of HF. The results indicate that the increase of sUA may be an important risk factor for the incidence and prognosis of HF (Huang et al., 2014; Vaduganathan et al., 2014; Palazzuoli et al., 2017; Pavlusova et al., 2019).

However, the mechanism of hyperuricemia-induced HF and its prognosis is not clear. Inflammation and oxidative stress play key roles in the development and progression of HF. XO is the key enzyme responsible for conversion of purine bases to UA and represents the major source of ROS production in circulation (Boban et al., 2014; Klisic et al., 2018). Therefore, the overactivation of XO may be important for the increase of mortality and hospitalization of patients with high UA and HF. Jia et al. found that the western diet resulted in increased sUA, cardiomyocyte hypertrophy, myocardial oxidative stress, myocardial fibrosis, and diastolic dysfunction (Jia et al., 2015). Further studies found that it may be related to the western diet enhancing the activation of S6 kinase-1 growth pathway and transforming growth factor-β1/Smad2/3 signal pathway and macrophage polarization (Jia et al., 2015). The latest research found that Vericiguat, a stimulator of soluble guanylate cyclase, treatment for 12 weeks, significantly decreases in hs-CRP and sUA in HF patients with reduced ejection fraction (Kramer et al., 2020). In addition, our research team also found that high UA stimulates ROS production, inhibits insulin-induced glucose uptake, and thus, leads to myocardial insulin resistance in H9c2 and primary cultured cardiomyocytes (Zhi et al., 2016; Li et al., 2018). Insulin resistance can inhibit the uptake of myocardial glucose, impairing lipid metabolism, leading to myocardial energy metabolism disorder, and thus, affecting the diastolic and contractile function of myocardium. Therefore, high UA-induced myocardial insulin resistance may be an important pathological mechanism of HF. Further study on the molecular mechanism of high UA-induced myocardial insulin resistance may be a novel target for intervention of hyperuricemic-related cardiovascular diseases.

Taken together, the increase of sUA is closely related to the occurrence and prognosis of HF. Inflammation and oxidative stress play key roles in the development and progression of HF. We suggest for the first time that high UA-induced insulin resistance may be an important pathological mechanism of HF. The relationship between high UA-induced myocardial insulin resistance and HF and whether ULT can improve the clinical outcome of patients with HF need to be proved in future studies (**Figure 3D**).

## Urate-lowering Treatments (ULT)

Currently, there are two main classes of ULT drugs commonly used in clinical practice: those inhibiting UA synthesis (XO inhibitors, such as allopurinol, febuxostat, etc.) and increasing UA excretion (e.g., benzbromarone, probenecid, etc.). Current studies confirm that ULT has a good effect in young hypertensive patients, but the effect on CHD, AF, and HF has not achieved satisfactory clinical results. In terms of drug selection, previous studies have suggested that febuxostat is more effective and safer than allopurinol. Recent studies have shown that ULT made no difference in the occurrence of primary end-point events (composite of cardiovascular death, nonfatal MI, nonfatal stroke, or unstable angina with urgent revascularization). However, cardiovascular death appeared to be higher in the febuxostat group compared to the allopurinol group (White et al., 2018). Therefore, febuxostat is neither recommended as the first-line ULT in the latest gout management guidelines nor for the treatment of asymptomatic hyperuricemia. Numerous experimental and clinical studies have confirmed that allopurinol can reduce the incidence of all-cause death, MI, and congestive HF in patients with hyperuricemia (Struthers et al., 2002; MacIsaac et al., 2016; Singh et al., 2017; Nidorf and Jelinek, 2018; Singh and Cleveland, 2018). For drugs promoting the excretion of UA, benzbromarone is superior to probenecid in efficacy and safety. Urate transporter 1 and organic-anion transporter 4 inhibitors, new drugs promoting the excretion of UA, are in the clinical trial stage.

Notably, sodium glucose cotransporter 2 inhibitors (SGLT-2; dapagliflozin, empagliflozin, canagliflozin, etc.) can effectively reduce sUA level by accelerating the excretion of UA (Wilcox et al., 2018; Zhao et al., 2018; Xin et al., 2019). Dapagliflozin, the earliest SGLT-2 inhibitor, reduced the level of UA in a dose-dependent manner. Compared to placebo, dapagliflozin 2.5/5/10 mg can decrease serum UA by 30.9/36.3/47.6 μmol/L, respectively (Bailey et al., 2010). The UA-lowering effect of SGLT-2 inhibitors can be attributed to glucose transporter-9 (GLUT-9) isoform 2. SGLT-2 inhibitors increased glucose concentration in renal tubules, activated GLUT-9 in proximal tubules, promoted glucose transport to cells, and excreted UA, and high glucose in collecting tubules inhibited the reabsorption of UA, both of which increased the excretion of UA. Recently, many clinical studies and meta-analyses confirmed that SGLT-2 inhibitors can reduce main cardiovascular adverse events, HF admission rate, and all-cause mortality in patients with hyperuricemia (Zinman et al., 2015; Neal et al., 2017; Wilcox et al., 2018; Zhao et al., 2018; McMurray et al., 2019; Wiviott et al., 2019; Xin et al., 2019). Therefore, SGLT-2 inhibitors are particularly suitable for reducing the risk of cardiovascular death in patients with T2D with hyperuricemia.

## Conclusions and Perspectives

Collectively, the relationship between hyperuricemia and cardiovascular disease is becoming clearer, which is attributed to the study progress of UA. First, Tomiyama et al. found that hyperuricemia increased arterial stiffness and inflammation, which may be involved in the risk of development of hypertension. Second, UA induces NLRP3 inflammasome-dependent inflammation activation via the AMPK-mTOR-mROS and HIF-1α pathways. In contrast, decrease of UA level promotes the activation of AMPK and inhibits the formation of atherosclerotic plaque. Furthermore, UA significantly enhances the expression of Kv1.5 protein and enhances Akt and HSF1 phosphorylation, leading to increased Hsp70 expression. This result suggests that inhibition of the Akt-HSF1-Hsp70 pathway may be a novel therapeutic approach against AF in patients with hyperuricemia. Last but not least, high UA induces insulin resistance in cardiomyocytes through the ROS-IRS1/Akt phosphorylation pathway. High UA-induced insulin resistance may be an important pathological mechanism of HF.

A large number of studies have shown that UA levels are positively correlated with hypertension, CHD, AF, and HF (**Table 1**); however, there was no satisfactory clinical outcome in the ULT of these cardiovascular diseases. Most clinical studies show that febuxostat improves cardiovascular outcomes in patients with gout, but whether it is as effective as allopurinol remains controversial. Allopurinol may increase the risk of serious adverse reactions, so it has not been widely used in clinical practice. Fortunately, SGLT-2 inhibitors (dapagliflozin, etc.) have a good prospect in ULT, meanwhile reducing main cardiovascular adverse events, HF admission rate, and all-cause mortality in patients with T2D and hyperuricemia. However, all of these need to be further confirmed by more large-scale clinical randomized controlled trials.

**Table 1:** Pivotal clinical studies on the relationship between sUA level and cardiovascular disease.

Study	Study design	Population	No. of Subjects	Main findings/outcomes
**Hypertension**				
Kuwabura et al., 2018b	Retrospective cohort	Prehypertension	3584	Increased sUA is a strong risk marker for developing hypertension from prehypertension
Sun et al., 2015	Prospective randomized	Adolescents	5478	A high level of UA indicated a higher likelihood of developing hypertension
Grayson et al., 2011	Meta-analysis (prospective)	Without hypertension	55607	Hyperuricemia is associated with an increased risk for incident hypertension, independent of traditional hypertension risk factors
Bjornstad et al., 2019	Follow-up RCT	Obese youth with T2D	539	Higher baseline sUA independently increased the risk for onset of hypertension
Tomiyama et al., 2018	Prospective	Men without hypertension	3274	Hyperuricemia may have a longitudinal association with the development of hypertension
Chen et al., 2018	Double-blind placebo RCT	Early Parkinson’s disease patients	75	Elevated urate is not association with high BP
Johnson et al., 2019	RCT	Chronic refractory gout	212	ULT can significantly reduce BP
**Coronary heart disease**				
Braga et al., 2016	Meta-analysis (prospective)	Without CVD	457915	Hyperuricemia appears to increase the risk of CHD events in the general population
Dai et al., 2015	Retrospective	Under 45 years old diagnosed with EOCAD	786	sUA >8 mg/dl was independently associated with triple branches involvement, HF and LV enlargement
von Lueder et al., 2015	Meta-analysis	Four clinical trials	12677	Elevated sUA is associated with poor outcomes
Li M. et al., 2016	Systematic review (prospective)	Hyperuricemia or elevated sUA level	958410	Hyperuricemia was associated with increased risk of CHD morbidity and mortality
Wang R. et al., 2016	Meta-analysis	With suspected or definite CHD	25229	Elevated sUA levels are strongly and independently associated with greater risk of cardiovascular and all-cause mortality
Jun et al., 2018	Retrospective cohort	CT evaluation of CAC	9297	sUA was an independent predictor for development of moderate CAC in subjects with no or minimal calcification
**Atrial fibrillation**				
Kuwabara et al., 2017c	Retrospective	Without general cardiovascular risk	49292	Hyperuricemia is an independent competing risk factor for AF
Zhang et al., 2016	Meta-analysis (prospective)	Hyperuricemia	426159	Hyperuricemia is associated with increased risk of AF
Pak et al., 2018	Meta-analysis	With and without AF	30609	The mean SUA level of patients with AF significantly is higher than those without AF
Canpolat et al., 2014	Prospective	Paroxysmal AF undergoing cryoablation	363	sUA levels were associated with a higher rate of AF recurrence
Zhao et al., 2016	Meta-analysis	AF undergoing cryoablation	1298	Elevated sUA is not associated with increased risk of AF recurrence after catheter ablation
**Heart failure**				
Pavlusova et al., 2019	Prospective	Acute HF	3610	Hyperuricemia was associated with an unfavorable cardiovascular risk
Palazzuoli et al., 2017	Prospective	Acute HF	324	Hyperuricemia was the only independent predictor of HF hospitalization or death
Vaduganathan et al., 2014	Double-blind placebo RCT	Worsening chronic HF	3955	sUA is commonly elevated in patients hospitalized for worsening chronic HF
Huang et al., 2014	Meta-analysis	Chronic HF	427917	Elevated sUA is associated with an increased risk of incident HF and adverse outcomes in HF patients

Numerous clinical studies and meta-analysis have shown that UA levels are significantly positively correlated with cardiovascular diseases, including hypertension, coronary atherosclerosis, AF, and HF.

AF, atrial fibrillation; BP, blood pressure; CAC, coronary artery calcium; CHD, coronary heart disease; CT, computed tomography; CVD, cardiovascular disease; EOCAD, early-onset coronary artery disease; HF, heart failure; LV, left ventricle; RCT, randomized controlled trial; sUA, serum uric acid; T2D, type 2 diabetes; UA, uric acid; ULT, uric acid-lowering treatments.

## Author Contributions

WY initiated this review, read lots of literature, and wrote the manuscript. J-DC revised our first draft and provided valuable comments. All authors contributed to the article and approved the submitted version.

## Funding

This work was supported by grants from the National Natural Science Foundation of China (81570772 and 81471081), the Natural Science Foundation of Fujian Province (2020J01010) and the Xiang’an Hospital of Xiamen University (PM201809170002).

## Conflict of Interest

The authors declare that the research was conducted in the absence of any commercial or financial relationships that could be construed as a potential conflict of interest.
